# Supplementary material for: X chromosome dosage drives statin-induced dysglycemia and mitochondrial dysfunction
Source: Nat Commun. 2024 Jul 2;15:5571. doi: 10.1038/s41467-024-49764-2 (PMC11219728; doi:10.1038/s41467-024-49764-2)
Supplement: Supplementary file 1 — Supplementary Information [file 41467_2024_49764_MOESM1_ESM.pdf]

Supplemental Data for Zhang et al. "X chromosome dosage drives statin-induced dysglycemia and mitochondrial dysfunction"

Supplementary Table 1: Gene expression and genotyping PCR primers

Gclc (forward)	5'- TGCACATCTACCACGCAGTCAA -3'
Gclc (reverse)	5'- TCAAGAACATCGCCTCCATTCA -3'
Glud1 (forward)	5'- GCAACCATGTGTTGAGCCTCT -3'
Glud1 (reverse)	5'- CCACAGCGCACTTGTATGTCA -3'
Got1 (forward)	5'- CGCCTAGTTCTTGGGGACAAC-3'
Got1 (reverse)	5'- TCCCAGGTTGGTGATGATACG -3'
Fasn (forward)	5'- GTTGGCCCAGAACTCCTGTA -3'
Fasn (reverse)	5'- GTCGTCTGCCTCCAGAGC -3'
Acaca (forward)	5'- GCCTCTTCCTGACAAACGAG -3'
Acaca (reverse)	5'- TGACTGCCGAAACATCTCTG -3'
Elovl6 (forward)	5'- GATGACCAAAGGCCTGAAGC -3'
Elovl6 (reverse)	5'- GTGGTGGTACCAGTGCAGGA -3'

Genotyping primers

Tg Sry (forward)	5'- AGCCCTACAGCCACATGATA -3'
Tg Sry (reverse)	5'- GTCTTGCCTGTATGTGATGG -3'
Ymt (forward)	5'- CTGGAGCTCTACAGTGATGA -3'
Ymt (reverse)	5'- CAGTTACCAATCAACACATCAC -3'
Myo (forward)	5'- TTACGTCCATCGTGGACAGCAT -3'
Myo (reverse)	5'- TGGGCTGGGTGTTAGTCTTAT -3'
oIMR0180	5'- GCCTAGCCGAGGGAGAGCCG -3'
oIMR0181	5'- TGTGACTTGGGAGCTCTGCAGC -3'
oIMR0182	5'- GCCGCCCCGACTGCATCT -3'

Supplementary Table 2: Summary demographics and clinical characteristics of individuals from whom iPSCs were developed

	Male		Female	
	Control	Case	Control	Case
**Population**				
Total Count	3	3	3	3
Age[1] (mean ± SD)	63.00 ± 7.25	59.00 ± 3.55	62.67 ± 8.73	67.67 ± 6.18
Race/Ethnicity	White			
**Statin Type - Initial**				
Lovastatin (%)	66.7	66.7	66.7	66.7
Simvastatin (%)	33.3	33.3	33.3	33.3
DDD[2] (mean ± SD)	0.83 ± 0.23	1.00 ± 0.00	0.92 ± 0.77	1.00 ± 0.70
mg/day (mean ± SD)	26.67 ± 9.42	33.33 ± 9.42	23.33 ± 12.4	26.67 ± 9.42
**Statin Type at Time of max FG**				
Lovastatin (%)	100	33.3	33.3	66.7
Simvastatin (%)	0	66.7	66.7	33.3
DDD (mean ± SD)	0.83 ± 0.23	1.67 ± 0.47	1.17 ± 0.62	1.33 ± 0.47
mg/day (mean ± SD)	33.33 ± 9.42	40.00 ± 0.00	26.67 ± 9.42	40.00 ± 0.00
**Pre-Statin**				
Fasting Glucose (mean ± SD)	99.33 ± 2.05	102.17 ± 3.56	88.33 ± 2.62	101.33 ± 9.42
Total Cholesterol (mean ± SD)	228.67 ± 39.63	219.17 ± 39.54	232.33 ± 34.45	252.00 ± 11.77
LDL (mean ± SD)	157.33 ± 39.74	119.00 ± 22.55	165.00 ± 36.24	162.67 ± 3.29
HDL (mean ± SD)	39.00 ± 4.32	34.00 ± 1.63	42.67 ± 5.43	51.33 ± 7.76
Triglyceride (mean ± SD)	162.00 ± 21.22	330.00 ± 257.68	122.67 ± 18.26	190.00 ± 22.37 *
**On-statin**				
Fasting Glucose (mean ± SD)	98.33 ± 0.47	139.33 ± 1.69 ****	87.67 ± 6.01	141.00 ± 39.75
Total Cholesterol (mean ± SD)	154.33 ± 10.20	164.67 ± 30.81	187.00 ± 36.36	207.00 ± 8.16
LDL (mean ± SD)	87.00 ± 7.48	69.67 ± 9.67	106.33 ± 29.04	122.33 ± 5.18
HDL (mean ± SD)	40.00 ± 2.94	33.67 ± 0.47 *	51.67 ± 9.39	54.67 ± 4.98
Triglyceride (mean ± SD)	138.33 ± 16.53	399.67 ± 333.72	144.67 ± 9.97	151.67 ± 14.61
**Delta**				
Fasting Glucose (mean ± SD)	-1.00 ± 2.44	37.17 ± 3.17 ***	-0.67 ± 3.39	39.67 ± 32.49
Total Cholesterol (mean ± SD)	-74.33 ± 30.30	-54.50 ± 19.47	-45.33 ± 11.44	-45.00 ± 8.48
LDL (mean ± SD)	-70.33 ± 32.83	-49.33 ± 16.04	-58.67 ± 16.04	-40.33 ± 7.40
HDL (mean ± SD)	1.00 ± 5.35	-0.33 ± 1.24	9.00 ± 5.65	3.33 ± 3.09
Triglyceride (mean ± SD)	-23.67 ± 6.12	69.67 ± 81.97	22.00 ± 13.63	-38.33 ± 12.03 **

* $P < 0.05$, ** $P < 0.01$, *** $P < 0.001$, **** $P < 0.0001$ case vs. control.

[1] Age at statin initiation

[2] DDD, defined daily dose

[3] Pre-statin clinical characteristics are vales taken closest and prior to statin initiation

[4] On-statin clinical characteristics are values taken around the time of maximum fasting glucose during the 3 years after statin initiation

[5] Delta are calculated as on-statin values minus pre-statin values.

Supplemental Figure 1

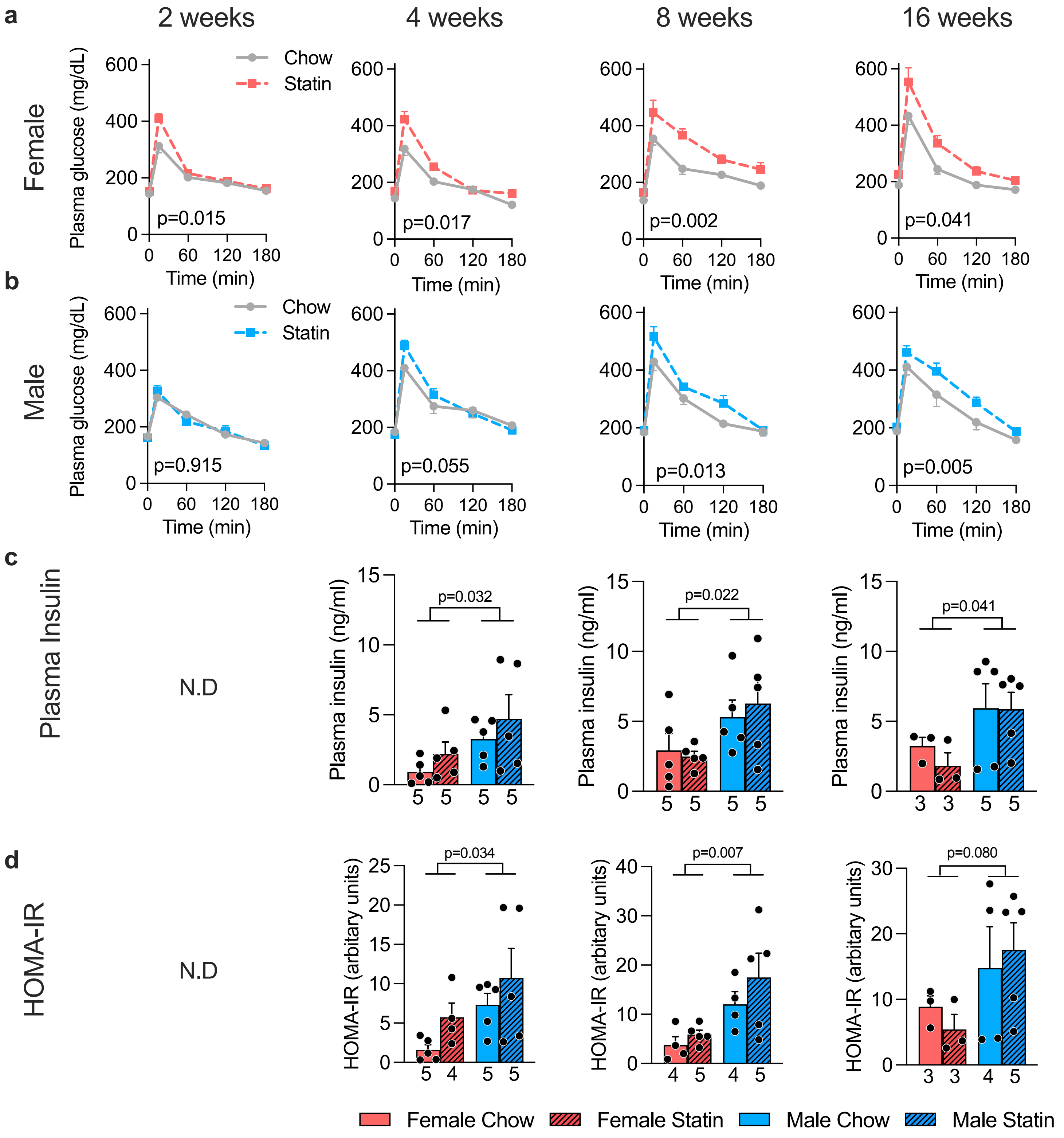

Suppl. Fig. 1. Glucose tolerance curves, plasma insulin, and HOMA-IR in chow and statin-fed female and male C57BL/6J mice. (a–b) Glucose tolerance curves for mouse cohorts shown in Fig. 1 d,e. N=5 for each sex and treatment. Data points are mean ± SEM. Area under the curve was compared by two-sided t-test and p values shown for control vs. statin treated animals on each graph. **(c)** Plasma insulin levels and **(d)** HOMA-IR in mice from Fig. 1d,e are shown as mean ± SEM. Data in (c) and (d) were analyzed by 2-way ANOVA, and where significant, subsequent pair-wise comparisons were performed via unpaired two-sided t-test. N for each group is indicated under bars. Source data are provided as a Source Data file.

Supplemental Figure 2

a

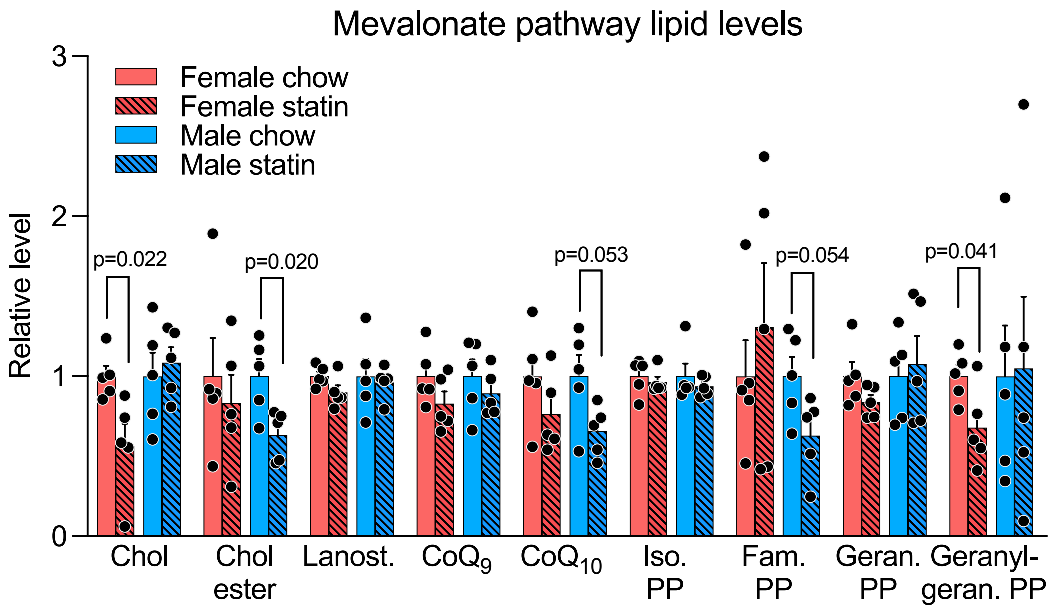

b

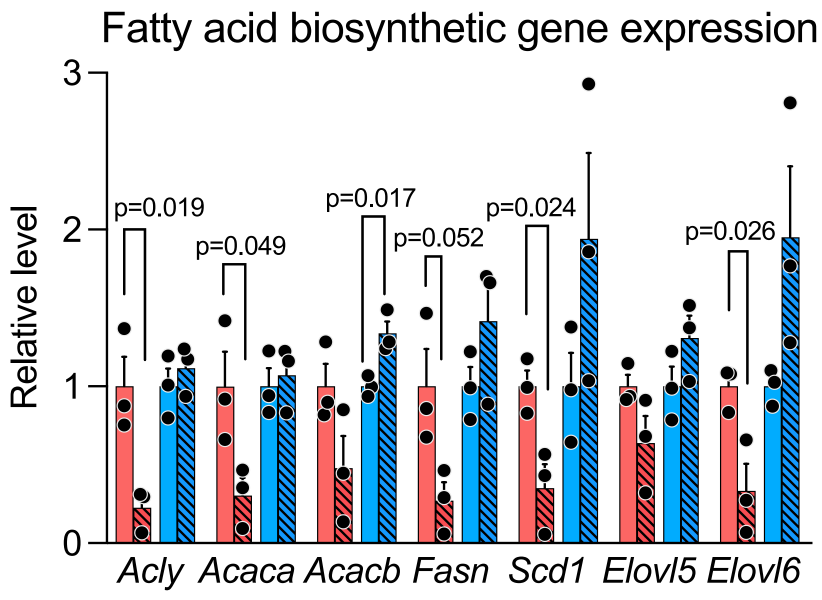

Suppl. Fig. 2. Sex differences in statin effects on cholesterol pathway intermediates and fatty acid biosynthetic gene expression. (a) Levels of hepatic lipids within the mevalonate pathway in male and female mice fed chow ± statin for 4 weeks (N=5mice/sex and treatment). Data are represented as mean ± SEM. Brackets show significant unpaired two-sided t-tests with each sex for specific lipids. Chol, cholesterol; Lanost., lanosterol; CoQ, coenzyme Q; Iso PP, isopentenyl pyrophosphate; Farn. PP, farnesyl pyrophosphate; Geran. PP, geranyl pyrophosphate; Geranyl geran. PP, geranylgeranyl pyrophosphate. **(b)** Statin-induced alterations in hepatic gene expression for fatty acid synthetic genes from mice shown in panel (a). *Acly*, ATC citrate lyase; *Acaca*, acetyl-CoA carboxylase; *Acacb*, acetyl-CoA carboxylase; *Fasn*, fatty acid synthase; *Scd1*, stearoyl-CoA desaturase 1; *Elovl5*, fatty acid elongase 5; *Elovl6*, fatty acid elongase 6. Values represent mean ± SEM. Brackets show significant unpaired two-sided t-tests with each sex for specific mRNAs. Source data are provided as a Source Data file.

Supplemental Figure 3

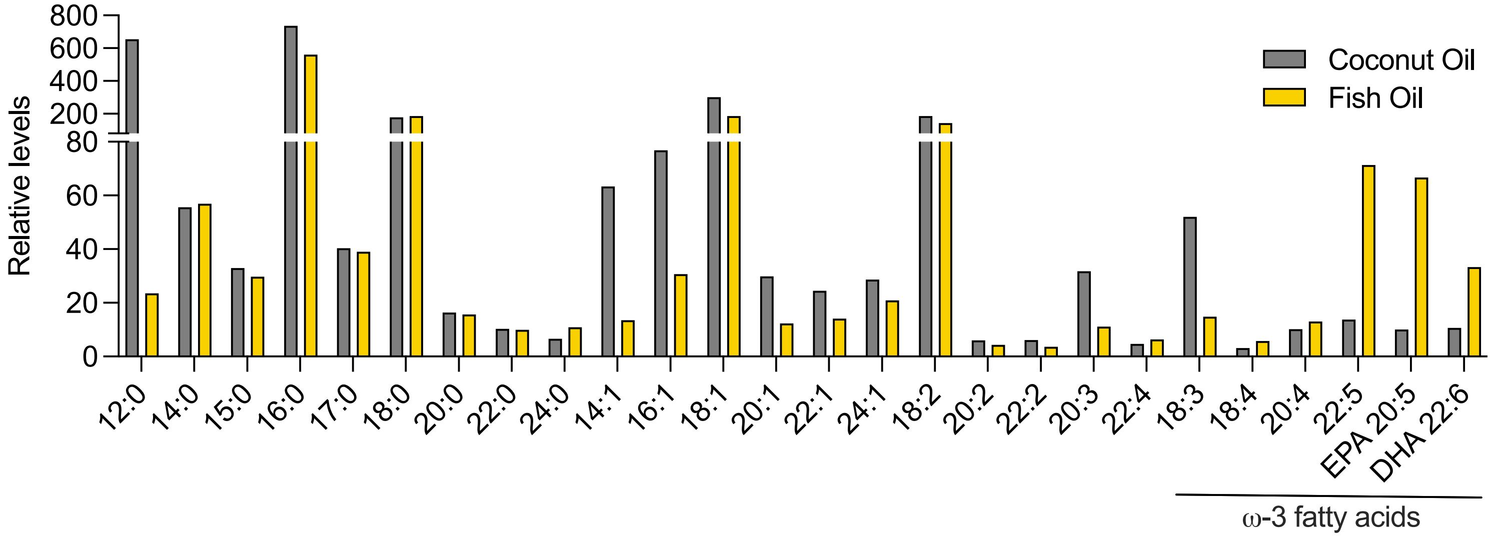

Suppl. Fig. 3. Fatty acid composition of coconut oil and fish oil used for in vivo studies shown in Figs 2 and 3. Lipidomic analysis was performed by mass spectrometry and fatty acids are denoted by carbon length:number of double bonds. The ω-3 fatty acids are grouped at right. Source data are provided as a Source Data file.

Supplemental Figure 4

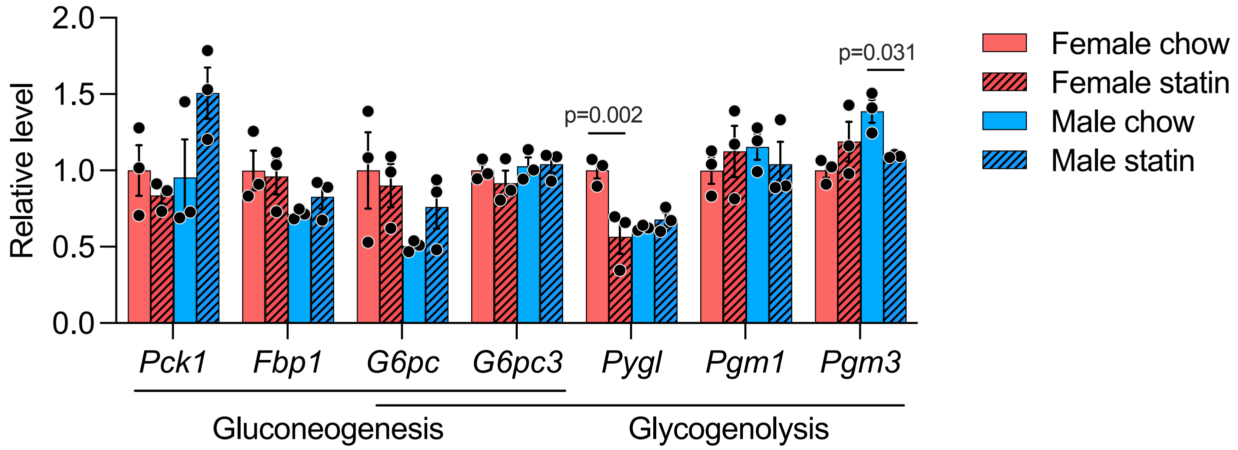

Suppl. Fig. 4. Gluconeogenesis and glycogenolysis pathway gene expression. Relative mRNA levels for genes denoted for the two pathways in male and female mice on chow ± statin for 4 weeks. Overlapping lines denote genes with roles in both gluconeogenesis and glycogenolysis. Values represent mean ± SEM. N=3 mice/sex and treatment. Significance determined by 2-way ANOVA followed by unpaired two-sided t-test. *Pck1*, phosphoenolpyruvate carboxykinase 1; *Fbp1*, fructose-bisphosphatase 1; *G6pc* and *G6pc3*, mouse glucose-6-phosphatase catalytic subunits; *Pygl*, glycogen phosphorylase; *Pgm1* and *Pgm3*, phosphoglucomutases. Source data are provided as a Source Data file.

Supplemental Figure 5

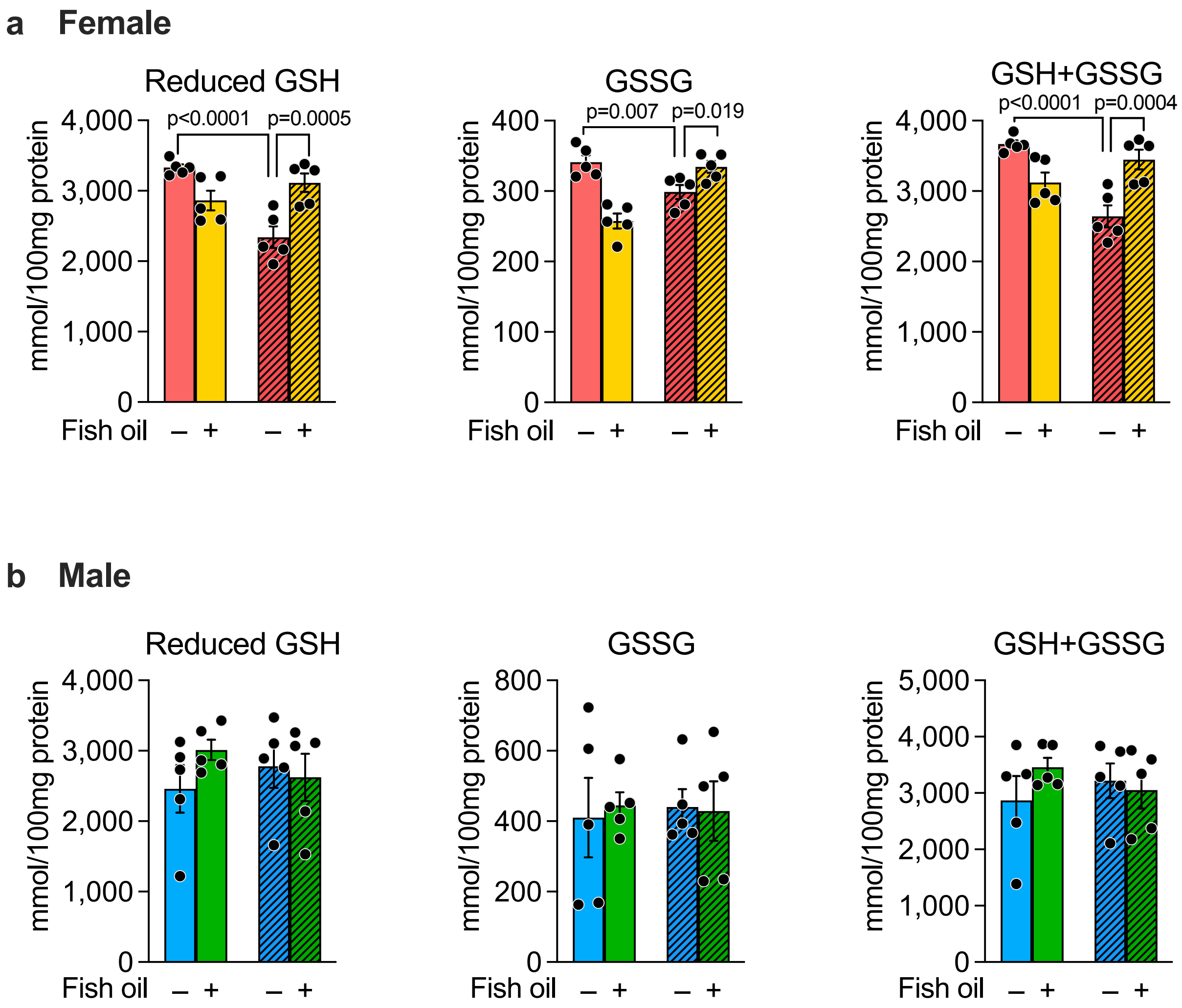

Suppl. Fig. 5. Glutathione levels in mice shown in Fig. 4g and i. Levels of reduced glutathione (GSH), oxidized glutathione (GSSG), and the ratio of reduced to oxidized glutathione (GSH/GSSH) were determined in **(a)** female and **(b)** male mice on each treatment (N=5/sex and treatment). Open bars, chow diet; striped bars, chow + statin diet. Data were analyzed by 2-way ANOVA and, where significant, followed by unpaired two-sided t-tests. Brackets represent significance of pairwise comparisons by t-test. Source data are provided as a Source Data file.

Supplemental Figure 6

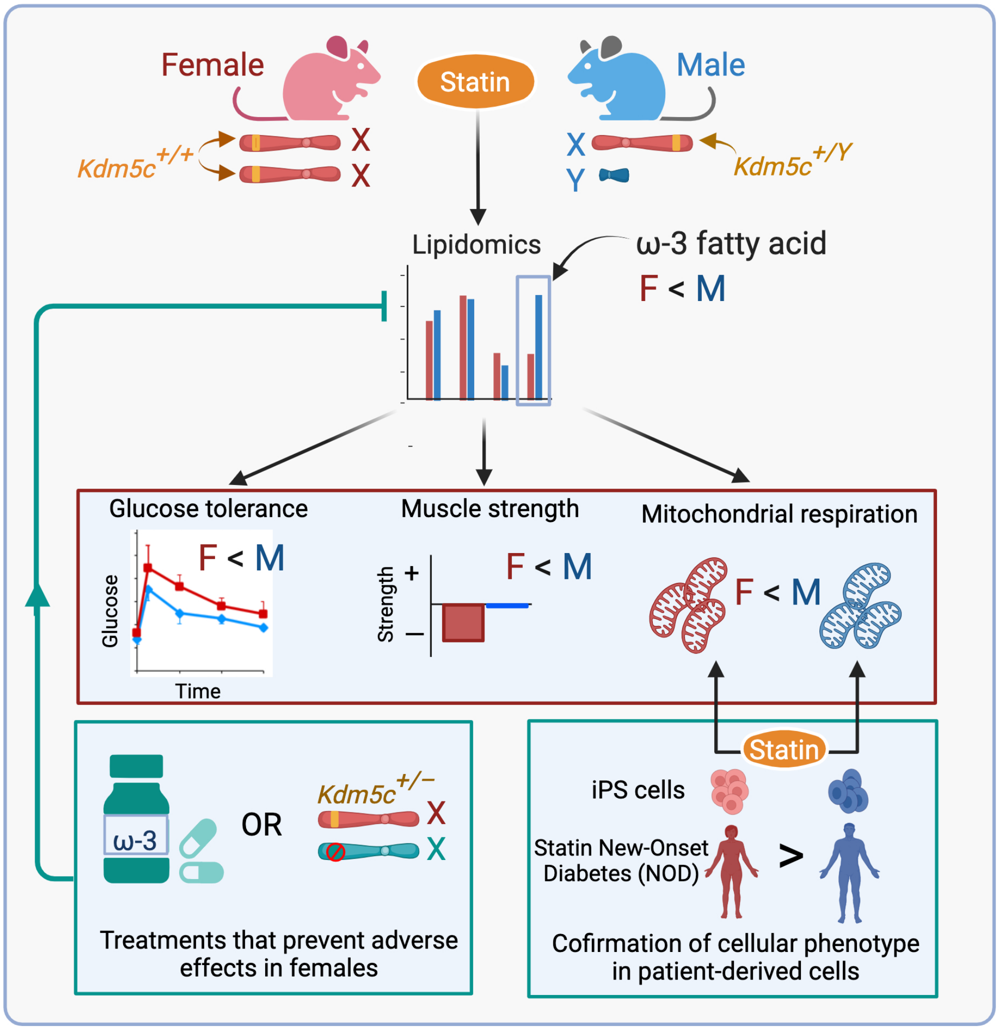

Suppl. Fig. 6. Graphical representation of the findings of sex differences in statin adverse effects in the mouse, their correction by ω-3 fatty acid administration or genetic reduction in *Kdm5c* gene dosage, and a validation of sex-biased statin impairment in cellular mitochondrial complex activity in human iPSCs from women that developed statin-related new-onset diabetes. Diagram was created with BioRender.com released under a Creative Commons Attribution-NonCommercial-NoDerivs 4.0 International License.
