# Supplementary material for: X chromosome dosage drives statin-induced dysglycemia and mitochondrial dysfunction
Source: Nat Commun. 2024 Jul 2;15:5571. doi: 10.1038/s41467-024-49764-2 (PMC11219728; doi:10.1038/s41467-024-49764-2)
Supplement: Supplementary file 3 — Description of Additional Supplementary Files [file 41467_2024_49764_MOESM3_ESM.pdf]

DESCRIPTION OF ADDITIONAL SUPPLEMENTARY FILES DOCUMENT

Supplementary Data 1 - Metabolomics data in Excel

Supplementary Data 2 - iPSC origin tables
