# Supplementary material for: X chromosome dosage drives statin-induced dysglycemia and mitochondrial dysfunction
Source: Nat Commun. 2024 Jul 2;15:5571. doi: 10.1038/s41467-024-49764-2 (PMC11219728; doi:10.1038/s41467-024-49764-2)
Supplement: Supplementary file 6 — Reporting Summary [file 41467_2024_49764_MOESM6_ESM.pdf]

nature portfolio

Corresponding author(s): Karen Reue

Last updated by author(s): 2024/2/6

Reporting Summary

Nature Portfolio wishes to improve the reproducibility of the work that we publish. This form provides structure for consistency and transparency in reporting. For further information on Nature Portfolio policies, see our Editorial Policies and the Editorial Policy Checklist.

Statistics

For all statistical analyses, confirm that the following items are present in the figure legend, table legend, main text, or Methods section.

n/a	Confirmed	
☐	☒	The exact sample size (*n*) for each experimental group/condition, given as a discrete number and unit of measurement
☐	☒	A statement on whether measurements were taken from distinct samples or whether the same sample was measured repeatedly
☐	☒	The statistical test(s) used AND whether they are one- or two-sided<br>*Only common tests should be described solely by name; describe more complex techniques in the Methods section.*
☐	☒	A description of all covariates tested
☒	☐	A description of any assumptions or corrections, such as tests of normality and adjustment for multiple comparisons
☐	☒	A full description of the statistical parameters including central tendency (e.g. means) or other basic estimates (e.g. regression coefficient) AND variation (e.g. standard deviation) or associated estimates of uncertainty (e.g. confidence intervals)
☐	☒	For null hypothesis testing, the test statistic (e.g. *F*, *t*, *r*) with confidence intervals, effect sizes, degrees of freedom and *P* value noted<br>*Give P values as exact values whenever suitable.*
☒	☐	For Bayesian analysis, information on the choice of priors and Markov chain Monte Carlo settings
☒	☐	For hierarchical and complex designs, identification of the appropriate level for tests and full reporting of outcomes
☒	☐	Estimates of effect sizes (e.g. Cohen's *d*, Pearson's *r*), indicating how they were calculated

Our web collection on statistics for biologists contains articles on many of the points above.

Software and code

Policy information about availability of computer code

Data collection	Real-time qPCR data was collected with Bio-Rad CFX Maestro v1.1 software. Mitochondrial respiration data were collected with Seahorse XF96 Analyzer v.1.4.2 (Agilent). Some grip-strength measurements were conducted with a custom apparatus that we constructed with an Arduino microcontroller that interfaces with OSX/Windows (Munier et al. (2022) Sci Rep 12:16428).
Data analysis	Statistical tests were performed with Prism v. 10.0 (GraphPad Software)<br>Volcano plots were generated with ggplot v3.3.6<br>Differential gene expression was analyzed with EdgeR v3.28.1<br>Respirometry data were analyzed using Seahorse XF96 Analyzer v1.4.2 (Agilent)<br>Pathway analysis of differential gene expression data was performed with Enrichr (https://maayanlab.cloud/Enrichr/#)

For manuscripts utilizing custom algorithms or software that are central to the research but not yet described in published literature, software must be made available to editors and reviewers. We strongly encourage code deposition in a community repository (e.g. GitHub). See the Nature Portfolio guidelines for submitting code & software for further information.

Data

Policy information about availability of data

All manuscripts must include a data availability statement. This statement should provide the following information, where applicable:

- Accession codes, unique identifiers, or web links for publicly available datasets
- A description of any restrictions on data availability
- For clinical datasets or third party data, please ensure that the statement adheres to our policy

Source data are provided with this paper for Figures 1–8 and Suppl. Fig. 1–5. Complete Western blots for Fig. 3c,d are provided in Source Data file. RNA-seq data associated with Fig. 2 have been deposited in GEO (Accession number GSE184588). Lipidomics data associated with Fig. 2 are provided as Suppl. Table 1.

Research involving human participants, their data, or biological material

Policy information about studies with human participants or human data. See also policy information about sex, gender (identity/presentation), and sexual orientation and race, ethnicity and racism.

Reporting on sex and gender	Our study uses blood samples that were previously obtained from men and women, who self-reported their sex (Abbasi et al. 2021 ATVB 41:2786–2797). Male and female sex is specified in all aspects of the study including data analyses, which are disaggregated by sex. Our study also uses iPSCs that were derived from human subjects and their self-reported sex is included in our data. No information on gender of donors used in the study were available, and we do not consider gender as a variable.
Reporting on race, ethnicity, or other socially relevant groupings	Samples from subjects are not grouped by race, ethnicity, or other social labels.
Population characteristics	Population characteristics for blood samples for DHA determinations: volunteers who were eligible for statin therapy for cardiovascular disease prevention and did not have type 2 diabetes, statin intolerance, or other exclusion criteria detailed in Abbasi et al. (2021 ATVB 41:2786–2797). Population characteristics for iPSC cell lines: Kuang et al. (2019 Stem Cell Res 37:101434) used electronic health records from Kaiser Permanente of Northern California to identify statin users who developed new-onset diabetes (NOD). NOD cases were defined as those who had his/her first statin (simvastatin, lovastatin, atorvastatin, pravastatin, rosuvastatin, or pitavastatin; Table S3) prescription at age 40–75, and documented continuous statin use for 3 years. Continuous statin use was defined as having greater than 8 30-day prescription refills per year or greater than 3 90-day prescription refills per year. Individuals prescribed statin combinations or with evidence of diabetes within the first 3 months of statin use were excluded. Additional inclusion/exclusion criteria are detailed in the Methods section of the manuscript.
Recruitment	Samples assessed here were collected for previously published studies. Blood samples for assessment of DHA levels were obtained from volunteers who were eligible for statin therapy for cardiovascular disease prevention and did not have type 2 diabetes, statin intolerance, or other exclusion criteria provided written informed consent (Abbasi et al. 2021 ATVB 41:2786–2797). Blood samples for iPSC development were obtained from volunteers for the Pharmacogenomics of Statin Therapy study (Kuang et al. 2019 Stem Cell Res 37:101434).
Ethics oversight	Blood samples for analysis of DHA levels were obtained with approval by the Stanford University Institutional Review Board (IRB 33347) (Abbasi et al. 2021 ATVB 41:2786–2797). Patient-derived iPSCs were generated from blood samples obtained under Institutional Review Boards of Kaiser Permanente Northern California and the UCSF Benioff Children's Hospital (Kuang et al. 2019 Stem Cell Res 37:101434).

Note that full information on the approval of the study protocol must also be provided in the manuscript.

Field-specific reporting

Please select the one below that is the best fit for your research. If you are not sure, read the appropriate sections before making your selection.

☒ Life sciences ☐ Behavioural & social sciences ☐ Ecological, evolutionary & environmental sciences

For a reference copy of the document with all sections, see nature.com/documents/nr-reporting-summary-flat.pdf

Life sciences study design

All studies must disclose on these points even when the disclosure is negative.

Sample size	Sample sizes for studies of mice or mouse tissues were based on pilot studies showing that n=6-8 animals was sufficient to detect inter-sex variation <15% for relevant traits and/or to distinguish the sex genotypes at p<0.05. Sample size for human studies was based on data from investigators' previous investigations of statin responses and power analyses. We used human blood samples that had been obtained in a previous study (Abbasi et al. 2021 ATVB41:2786–2797). The number of subjects recruited for this study was 69, which was selected to allow detection of an 8% change in insulin resistance in response to statin therapy with 80% power and 2-sided significance level of 5% using a paired t-test. In our analysis, we segregated the subjects by sex (43 men, 26 women), and calculated that we could detect a differences of 30

points in DHA fatty acid levels at 80% power and 2-sided significance level of 5% using a paired t-test.

Data exclusions	No data were excluded.
Replication	We used 5 independent C57BL/6J mouse cohorts (n=5 mice/sex/cohort) to demonstrate that females have a more rapid development of adverse effects from statin treatment than males. We used 2 cohorts of fish oil or control treatment (n=5 per sex and each of 4 treatments). Experiments with Four Core Genotypes mice and Kdm5c++/Kdm5c+/– mice were performed with a single cohort. All replications were consistent.
Randomization	Animals of the same genotype were randomly allocated to control and treatment groups (for example, statin vs. control chow or vehicle and fish oil groups).
Blinding	For measurements of blood and tissue parameters (gene expression by RNA-seq, metabolomic analyses), experimenters were blinded to sample identity. For measurements in live mice (grip strength, glucose levels), individuals making measurements were handed mice in a random sequence by a separate experimenter. Analysis of human blood samples were performed in a blind manner.

Reporting for specific materials, systems and methods

We require information from authors about some types of materials, experimental systems and methods used in many studies. Here, indicate whether each material, system or method listed is relevant to your study. If you are not sure if a list item applies to your research, read the appropriate section before selecting a response.

Materials & experimental systems

n/a	Involved in the study
☐	☒ Antibodies
☐	☒ Eukaryotic cell lines
☒	☐ Palaeontology and archaeology
☐	☒ Animals and other organisms
☒	☐ Clinical data
☒	☐ Dual use research of concern
☒	☐ Plants

Methods

n/a	Involved in the study
☒	☐ ChIP-seq
☒	☐ Flow cytometry
☒	☐ MRI-based neuroimaging

Antibodies

Antibodies used	Anti-GSK-3b (#9315, clone 27C10, Cell Signaling Technology), anti-phospho-GSK-3b-Ser9 (#9336, lot 14, Cell Signaling Technology) , anti-GAPDH (GeneTex #GTX100118, lot unknown).
Validation	GAPDH antibody has been validated by GeneTex on numerous Western blots form and tissue slices from many tissue types and is cited in >1000 publications. Anti-GSK3b antibody was validated by Cell Signaling by Western blot and immunohistochemistry with and without blocking peptide, and cited in >1000 publications. Anti-phospho-GSK-3b Ser9 antibody was validated by Cell Signaling in the absence and presence of phosphatase and has been cited in >900 publications.

Eukaryotic cell lines

Policy information about cell lines and Sex and Gender in Research

Cell line source(s)	Hepa1-6 cells were from the American Type Culture Collection (ATCC CRL-1830). Human iPSC lines were generated as described in Kuang et al. (2019) Stem Cell Res 37:101434. Lines derived from both males and females were used and analyzed separately (sex determined by self-reporting by donor and by gene expression of sex dosage-dependent genes).
Authentication	Cell lines were authenticated as iPSCs using a panel of pluripotent and lineage specific markers and by differentiation in vitro to three germ layers (Kuang et al. 2019 Stem Cell Res 37:101434). Hepa1-6 cells were not authenticated.
Mycoplasma contamination	Cells were not tested for Mycoplasma contamination.
Commonly misidentified lines (See ICLAC register)	No commonly misidentified lines were used.

Animals and other research organisms

Policy information about studies involving animals; ARRIVE guidelines recommended for reporting animal research, and Sex and Gender in Research

Laboratory animals	Male and female C57BL/6J mice were obtained from The Jackson Laboratory (Bar Harbor, ME). C57BL/6 Four Core Genotypes mice with Apoe deficiency were bred at UCLA and have been previously backcrossed to a C57BL/6 background as reported (Wiese et al. (2022) Biol Sex Diff 13:63). Kdm5c+/+ and Kdm5c+/– mice were bred at UCLA and were previously backcrossed to C57BL/6J for 11 generations as described previously (Link et al. 2020 J Clin Invest 130:5688–5702). Mice were assigned to treatment groups at 8–10

	weeks of age and blood and tissues collected at specified times after treatment, ranging from 2 weeks to 16 weeks. Males and females were used for all studies except only female Kdm5c+/– mice were used because male Kdm5cY/–C57BL/6 mice are not viable.
Wild animals	No wild animals were used in the study.
Reporting on sex	Males and females were used for all studies of wild-type mice, and all data was analyzed after disaggregation by sex. Studies of the Four Core Genotypes Mice include 4 sex genotypes (XX with ovaries, XY with ovaries, XX with testes and XX with ovaries) and all genotypes were included an analysis performed with sex disaggregation. Studies of Kdm5c+/– mice were performed in females only because male Kdm5cY/–C57BL/6 mice are not viable.
Field-collected samples	No field collected samples were used in the study.
Ethics oversight	All studies were approved by the UCLA Institutional Animal Care and Use Committee.

Note that full information on the approval of the study protocol must also be provided in the manuscript.

Plants

Seed stocks	No seeds or plants were used in this study.
Novel plant genotypes	No seeds or plants were used in this study.
Authentication	No seeds or plants were used in this study.
